# ASO Author Reflections: The Pursuit of Digitalised Quality Improvement Assessment Tools for Cancer Multidisciplinary Teams

**DOI:** 10.1245/s10434-021-09988-8

**Published:** 2021-04-20

**Authors:** Benjamin W. Lamb, S. Miah, T. Soukup

**Affiliations:** 1 Department of Urology, Cambridge University Hospitals NHS Foundation Trust, Cambridge, UK; 2 School of Allied Health, Anglia Ruskin University, Cambridge, UK; 3 Department of Urology, Buckinghamshire Healthcare NHS Trust, Amersham, UK; 4 Health Service and Population Research Department, Center for Implementation Science, King’s College London, London, UK

## Past

For the past 20 years, the cornerstone of cancer care has been the multidisciplinary tumour board (MTB), who meet regularly to discuss care for people with cancer. MTBs are valued, but the quality of their functionality is variable and affected by a number of factors from logistics to team composition, complexity of cases, and time and workload pressures.^1^,^2^ Methods have been established for assessing and improving the way MTBs work in their meetings. A number of tools currently exist that focus on different aspects of MTBs’ functionality.^3^ One such example is the Metric for the Observation of Decision-making (MODe). This was developed with a focus on assessing and improving MTBs’ decision-making and teamwork, but it can be cumbersome for use in routine clinical practice by healthcare staff.^4^ We set out to modify the MODe tool so that it could more readily be used by MTBs interested in assessing and improving the way they work.^5^

## Present

The present study demonstrates good validity and reliability of the shorter version of MODe, namely MODe-LITE. It also shows that the learning curve for a novice user is shorter than for the original MODe tool. This implies that MODe-LITE could be used in routine clinical practice after a shorter period of training.^5^

## Future

Future work should aim to digitalise MODe-LITE and other assessment tools for MTBs so that they can be undertaken using electronic devices, such as a tablet or smartphone, and integrated with electronic medical records. Digitisation would facilitate data collection and analysis. Further research is needed to validate MTB assessment tools for virtual or remote MTB meetings, which are more prevalent since outbreak of the COVID-19 pandemic. Routine use of MTB assessment tools as part of quality improvement could improve decision-making and team working, ultimately translating to improved patient care.
